# Reduced Ets Domain-containing Protein Elk1 Promotes Pulmonary Fibrosis via Increased Integrin αvβ6 Expression*

**DOI:** 10.1074/jbc.M115.692368

**Published:** 2016-02-09

**Authors:** Amanda L. Tatler, Anthony Habgood, Joanne Porte, Alison E. John, Anastasios Stavrou, Emily Hodge, Cheryl Kerama-Likoko, Shelia M. Violette, Paul H. Weinreb, Alan J. Knox, Geoffrey Laurent, Helen Parfrey, Paul John Wolters, William Wallace, Siegfried Alberti, Alfred Nordheim, Gisli Jenkins

**Affiliations:** From the ‡ Division of Respiratory Medicine, University of Nottingham, Nottingham University Hospitals, City Campus, Nottingham NG5 1PB, United Kingdom,; § Biogen Inc., Cambridge, Massachusetts 02142,; the ¶ Centre for Respiratory Research, University College London, London WC1E 6JF, United Kingdom,; the ‖ Centre for Cell Therapy and Regenerative Medicine, University of Western Australia, Crawley WA 6009, Australia,; the ** Department of Medicine, University of Cambridge and Papworth Hospital NHSFT, Cambridge CB2 0SP, United Kingdom,; the ‡‡ Department of Medicine, University of California, San Francisco, San Francisco, California 94143,; the §§ Division of Pathology, University of Edinburgh, Edinburgh EH4 2XR, United Kingdom, and; the ¶¶ Interfaculty Institute of Cell Biology, Tübingen University, Tübingen 72076, Germany

**Keywords:** fibrosis, gene regulation, integrin, lung, pulmonary fibrosis, elk1

## Abstract

Idiopathic pulmonary fibrosis (IPF) is a progressive fibrotic lung disease with high mortality. Active TGFβ1 is considered central to the pathogenesis of IPF. A major mechanism of TGFβ1 activation in the lung involves the epithelially restricted αvβ6 integrin. Expression of the αvβ6 integrin is dramatically increased in IPF. How αvβ6 integrin expression is regulated in the pulmonary epithelium is unknown. Here we identify a region in the β6 subunit gene (*ITGB6*) promoter acting to markedly repress basal gene transcription, which responds to both the Ets domain-containing protein Elk1 (Elk1) and the glucocorticoid receptor (GR). Both Elk1 and GR can regulate αvβ6 integrin expression *in vitro*. We demonstrate Elk1 binding to the *ITGB6* promoter basally and that manipulation of Elk1 or Elk1 binding alters *ITGB6* promoter activity, gene transcription, and αvβ6 integrin expression. Crucially, we find that loss of Elk1 causes enhanced *Itgb6* expression and exaggerated lung fibrosis in an *in vivo* model of fibrosis, whereas the GR agonist dexamethasone inhibits *Itgb6* expression. Moreover, Elk1 dysregulation is present in epithelium from patients with IPF. These data reveal a novel role for Elk1 regulating *ITGB6* expression and highlight how dysregulation of Elk1 can contribute to human disease.

## Introduction

The prognosis of idiopathic pulmonary fibrosis (IPF)^2^ patients is poor, with 5-year survival rates worse than most cancers (1), and the incidence is rapidly increasing (2). IPF is characterized by excessive matrix deposition within the lung interstitium, leading to deteriorating pulmonary function and, ultimately, death. IPF pathogenesis is poorly understood; however, the current paradigm suggests that recurrent epithelial injury and failing repair promote expansion and trans-differentiation of myofibroblasts within the interstitium (3). An improved understanding of the mechanisms driving fibrogenesis is critical for the development of urgently needed new therapeutic agents.

TGFβ1 is a key cytokine implicated in the development of fibrosis (4). It has profound effects on both epithelial cells and fibroblasts, two of the key effector cells in fibrogenesis, and mediates many of the characteristic pathologic features of fibrosis, including epithelial cell apoptosis, myofibroblast trans-differentiation, and subsequent collagen production (5, 6). For TGFβ1 to exert any biological effect, it must first be activated, and activation by the αvβ6 integrin is an important feature in experimental models of fibrosis in multiple organ systems (7–12).

Fundamentally, high levels of αvβ6 integrins have been demonstrated in lung tissue from patients with pulmonary fibrosis (9, 13) and are associated with a worse prognosis (14). Similarly, levels of β6 subunit mRNA (*ITGB6*) correlate with increasing severity of fibrosis in the liver (15). The molecular mechanisms regulating the expression levels of αvβ6 integrins are, therefore, likely to be critical for ensuring normal repair in response to injury rather than pathological fibrosis. Such mechanisms, however, have not been investigated in detail.

It has been shown previously that TGFβ1 increases *ITGB6* levels and elevates expression of αvβ6 integrins by guinea pig airway epithelial cells *in vitro* (16), and a positive feedback loop of αvβ6-mediated TGFβ1 activation promoting enhanced integrin expression in the lung has been proposed (17). It is possible that TGFβ1-independent pathways may also contribute to *ITGB6* regulation. For example a potential role for ets-1 during increased transcription of the *ITGB6* gene has been suggested (18), and overexpression of active Stat3 leads to increased *ITGB6* expression in carcinogenesis (19). However, whether these mechanisms are involved in up-regulation of *ITGB6* in pulmonary fibrosis is unknown.

This study aimed to investigate the molecular mechanisms driving *ITGB6* expression in lung epithelial cells and investigate the importance of these pathways in fibrogenesis. We identified a novel region of the *ITGB6* promoter responsible for repressing transcription and demonstrate that Elk1, as well as the glucocorticoid receptor (GR), can act to repress the *ITGB6* gene. We demonstrate that Elk1 deficiency *in vivo* enhances *Itgb6* expression and exacerbates bleomycin-induced fibrosis in mice, whereas the GR agonist dexamethasone represses *Itgb6* expression. Finally, we show that lung tissue from pulmonary fibrosis patients displays reduced Elk1 expression associated with reduced Elk1 binding to the *ITGB6* promoter. These data highlight a novel and crucial function of the transcription factor Elk1 in modulating αvβ6 expression in the lung.

## Experimental Procedures

### 

#### 

##### Cell Lines and Reagents

Immortalized human bronchial epithelial cells (iHBECs, a gift from Prof. Jerry Shay, University of Texas) or small airway epithelial cells (SAECs, Lonza) were used for many *in vitro* experiments. The iHBEC immortalized cell line was selected over primary alveolar epithelial cells for the majority of *in vitro* studies because of the technical advantages in performing molecular mechanistic studies suchas transfections and ChIP in these cells. iHBECs retain their ability to differentiate into basal, mucin-producing, and ciliated epithelial cells (20). Furthermore, iHBECs are one of the only immortalized or transformed lung epithelial cell lines that retain their αvβ6 integrin expression *in vitro*. iHBECs were cultured in keratinocyte serum-free medium (Lonza) containing bovine pituitary extract (Lonza), epidermal growth factor (Lonza), G418 (Sigma-Aldrich), puromycin (Sigma-Aldrich), and penicillin and streptomycin (both from Sigma-Aldrich).

Primary SAECs were used to confirm some findings obtained in iHBECs. They were cultured in small airway growth medium (Lonza) containing the supplied supplements. All cells were growth-arrested in supplement-free medium for 24 h prior to the start of experiments, except in the case of transfection experiments. To assess binding of endogenous Elk1 to exogenous *ITGB6* promoter constructs, we used the adenosquamous carcinoma cell line H647 after determining that these cells exhibit a high expression level of endogenous Elk1. H647 cells were cultured in RPMI medium (Sigma-Aldrich) with 10% FCS, l-glutamine, penicillin, and streptomycin.

Elk1 and GAPDH antibodies for Western blotting were supplied by Cell Signaling Technology. All siRNA constructs and the associated reagents were provided by Santa Cruz Biotechnology. Formaldehyde, glycine, dexamethasone, and progesterone were supplied by Sigma-Aldrich. Phycoerythrin-labeled or FITC-labeled secondary antibodies for flow cytometry, TRIzol reagent, zysorbin, the TA cloning kit, and T4 ligase were supplied by Invitrogen. All reagents required for the synthesis of cDNA from RNA, including Moloney murine virus reverse transcriptase, and all plasmids not provided by Addgene or a collaborator plus Transfast reagent were provided by Promega. Kapa Taq polymerase for use in QPCR reactions was supplied by Kapa Biosystems. All ChIP antibodies were obtained from Abcam.

##### Flow Cytometry

Flow cytometry was used to assess αvβ6 cell surface expression as described previously (13). Briefly, iHBECs were labeled with 10 μg of anti-αvβ6 antibody (clone 6.3G9) (21) and a PE-labeled anti-mouse secondary antibody. A secondary antibody-only negative control was performed. Fluorescence was assessed using a BD FACSDIVA flow cytometer.

##### Quantitative PCR

Gene expression of both human and murine genes was measured using an MxPro3000 instrument (Stratagene) and Kapa Taq using the following primer sequences: human *ITGB6*, 5′-AAACGGGAACCAATCCTCTGT-3′ (sense) and 5′-GCTTCTCCCTGTGCTTGTAGGT-3′ (antisense);human β2-microglobulin (*B2M*), 5′-AATCCAAATGCGGCATCT-3′ (sense) and 5′-GAGTATGCCTGCCGTGTG-3′ (antisense); human *ELK1*, 5′-CCACCTTCACCATCCAGTCT-3′ (sense) and 5′-TCTTCCGATTTCAGGTTTGG-3′ (antisense);and murine *Itgb6*, 5′-TCTGAGGATGGAGTGCTGTG-3′ (sense) and 5′-GGCACCAATGGCTTTACACT-3′ (antisense). All QPCR reactions were performed at an annealing temperature of 60 °C. Amplification of a single DNA product was confirmed by melting curve analysis. Data were expressed as relative expression using the ΔΔCt equation.

##### Generation of the ITGB6 Promoter Deletion Mutant Series

**FIGURE 1.**
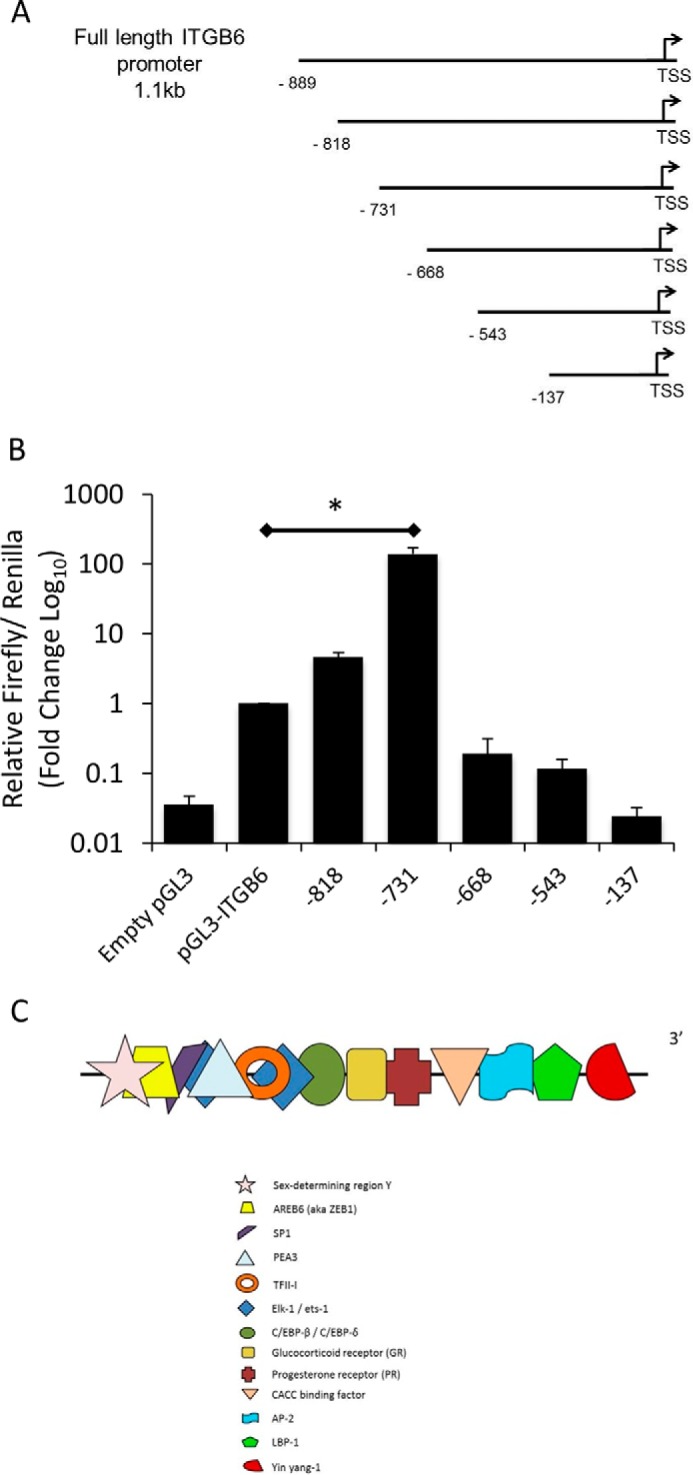
**The ITGB6 gene promoter contains a large repressor region.** *A*, schematic demonstrating the series of deletion mutants of the full-length pGL3-ITGB6 promoter luciferase reporter construct. *TSS*, transcription start site. *B*, iHBECs were transfected with the either empty pGL3 vector, full-length *ITGB6* promoter reporter, or a series of *ITGB6* promoter deletion mutants. After 4 h, luciferase activity was measured to determine promoter activity. Data are expressed as mean ± S.E. relative to the firefly/*Renilla* ratio from three independent experiments. *, *p* < 0.05. *C*, *in silico* analysis revealed the presence of several transcription factor binding sites between −818 and −731 from the transcription start site. This schematic shows these transcription factor binding sites.

The 1.1-kb insert was excised from pGL2-*ITGB6* (18) and ligated into the pGL3 vector (Promega) using T4 ligase and then sequenced to verify the presence of the insert. Progressively truncated forms of the reporter (Fig. 1*A*) were amplified by PCR and TA-cloned with a commercially available kit (Invitrogen). The purified inserts were then ligated into the pGL3 vector using T4 ligase (Invitrogen) and sequenced to confirm the presence of the correct insert.

##### Reporter Construct Transfections

Transient transfections were performed using Transfast (Promega) transfection reagent using 0.75 μg of reporter plasmid DNA with 7.5 ng of *Renilla* luciferase DNA at a 1:2 DNA:Transfast ratio. Briefly, cells were seeded at 2 × 10^5^ cells/ml and then cultured for 8 h in supplemented keratinocyte serum-free medium prior to transfection overnight in unsupplemented keratinocyte serum-free medium. The following day, cells were stimulated as required for the experiment. To assess the binding of endogenous Elk1 to *ITGB6* reporter constructs, 0.75 μg of reporter plasmid DNA was transfected in to H647 cells at a 1:2 DNA:Transfast ratio for 24 h.

##### siRNA Transfections

siRNA directed toward *Elk1, GR*, or a control siRNA were transfected into iHBECs using the siRNA transfection reagents and protocol provided by Santa Cruz Biotechnology. 40 pmol of siRNA was used per well of cells in a 24-well plate. Cells were transfected for 24 h prior to the start of an experiment to allow sufficient knockdown of target protein.

##### Site-directed Mutagenesis (SDM)

Site-directed mutagenesis was performed using the QuikChange II site-directed mutagenesis kit (Agilent) according to the instructions of the manufacturer. The Elk1 binding sites located at −772 (reverse strand) and −752 were mutated from GGAA to AAGA using the following primers: sense, 5′-CCCCCAGGGTGTTCTTCAAAGAGGTGTTTTGAGATTTGTAGAGTC-3′; antisense, 5′-GACTCTACAAATCTCAAAACACCTCTTTGAAGAACACCCTGGGGG-3′. All constructs were sequenced prior to use in experiments to confirm the presence of the correct mutation.

##### Chromatin Immunoprecipitation

ChIP allowed the binding of proteins to the *ITGB6* promoter to be determined. The ChIP-IT Express kit (Active Motif) was used to assess binding in cultured cells as described previously for airway smooth muscle cells (22). Briefly, cells were fixed with 1% formaldehyde for 5 min and lysed prior to shearing of the cellular chromatin by sonication using an Epishear sonicator (Active Motif). 25 μg of total chromatin was immunoprecipitated with 10 μg of antibody and protein G magnetic beads. ChIP QPCR data were normalized to input chromatin (*i.e.* the amount of total chromatin present in the immunoprecipitation sample) and then expressed as relative binding compared with the control sample using the ΔΔCt equation. ChIP QPCR primer sequences were as follows, using an annealing temperature of 60 °C: *ITGB6* promoter −934 to −753, 5′-CATGCTTACCCAGGAATGCT-3′ (sense) and 5′-ACACCCTGGGGGAAAAATAC-3′ (antisense); *ITGB6* promoter −1604 to −1497, 5′-ACTGCCCTCCAGCCTAGAA-3′ (sense) and 5′-TTGACAGAAACTAAATGCCCAA-3′ (antisense).

ChIP on human lung tissue was performed by first creating a single-cell suspension by passing the tissue through a 100-μm cell strainer. The cellular chromatin was then cross-linked in 1% formaldehyde for 5 min. The cross-linking was reversed with glycine, the cells were lysed in 10% SDS, and then the chromatin was sheared by sonication using an Epishear sonicator (Active Motif). 100 μg of total tissue chromatin was subjected to immunoprecipitation with 10 μg of Elk1 antibody overnight. Antibody-bound DNA was extracted using Zysorbin.

ChIP was employed to assess binding of endogenous Elk1 protein to exogenous *ITGB6* promoter plasmid constructs. H647 cells were used, which we found to express high levels of endogenous Elk1 (data not shown). Following transfection and expression of exogenous constructs, the cells were formaldehyde-fixed and processed as stated above for other cultured cells. QPCR of the resulting immunoprecipitated DNA was performed using primers directed against the luciferase sequence of the pGL3 plasmid to assess Elk1 binding only to exogenous DNA, not endogenous DNA (sense, 5′-GCTTTTACAGATGCACATATC-3′; antisense, 5′-CCATACTGTTGAGCAATTCACG-3′). Relative binding was normalized to the input chromatin luciferase critical threshold signal (*i.e.* the amount of luciferase plasmid DNA present) to ensure that differences in transfection efficiency between the constructs did not affect detection of Elk1 binding levels. Data were expressed as relative binding to the non-mutated *ITGB6* promoter construct using the ΔΔCt equation.

##### Western Blotting

Human lung tissue was homogenized using a mechanical tissue tearer in ice-cold lysis buffer (20 mm Tris-HCl, 137 mm NaCl, 1% Triton X-100, 2 mm EDTA, and 10% glycerol, all supplied by Sigma-Aldrich) supplemented with protease and phosphatase inhibitors (Complete Mini protease inhibitor tablet and Phos-stop inhibitor tablets, Roche). Expression of Elk1, GR, and GAPDH proteins was measured by Western blotting as described previously for Smad2/3 (23). Briefly, protein concentrations in each sample were determined using BCA (Pierce) according to the instructions of the manufacturer and using increasing concentrations of bovine serum albumin as a standard curve. 100 μg of total protein was loaded into each lane of an SDS-PAGE gel, and a current of 150 V was applied for 60 min. The gel was transferred onto a polyvinylidene membrane, blocked with 5% nonfat milk, and then probed with the following antibodies overnight: rabbit anti-Elk1 (Cell Signaling Technology, 9182) at 1:1000 dilution, rabbit anti-GR (Cell Signaling Technology, 3660) at 1:1000 dilution, and goat anti-GAPDH (V-18, Santa Cruz Biotechnology) at 100 ng/ml. All horseradish peroxidase-conjugated secondary antibodies were used at 333 μg/ml and were purchased from Dako. Western blotting films were scanned, and the band density was calculated by inverting the image and measuring mean gray pixels using Adobe Photoshop CS5 Extended (32-bit, version 12.1).

##### In Vivo Bleomycin Model of Pulmonary Fibrosis

Male *Elk1*^−/^*^0^* (24) and wild-type mice (aged 5–7 weeks) were treated with either 0 or 60 IU of bleomycin sulfate in 50 μl of saline via the oropharyngeal route under isoflurane anesthesia (2.5%, 2 liters/min flow of oxygen). All tissue was collected 28 days after bleomycin treatment. The lungs were either excised and snap-frozen at −80 °C for mRNA and hydroxyproline assessment or insufflated with formalin at constant gravitational pressure (20 cm H_2_O) and then paraffin wax-embedded for histology and immunohistochemistry.

To assess the role of GR in regulating *Itgb6* mRNA *in vivo*, male 6-week-old C57/black6/J mice were treated with either 60 of IU bleomycin in 50 μl of saline or saline only as a control via the oropharyngeal route under isoflurane anesthesia (2.5%, 2 liters/min). All animals also received daily treatment with either 1 mg/kg dexamethasone (100-μl volume) or PBS only as a control via intraperitoneal injection. After 14 days, the lungs were removed and snap-frozen at −80 °C for subsequent RNA analysis.

##### Immunohistochemistry

5-μm-thick sections of paraffin wax-embedded lung tissue of murine and human origin was subjected to immunohistochemistry to evaluate expression levels of αvβ6 integrin as described previously (13). An antibody directed against murine αvβ6 (clone ch2.A1) and an antibody directed against human αvβ6 (clone 6.3G9) were provided by Biogen Idec. Murine tissue from the bleomycin model of pulmonary fibrosis was also subjected to immunohistochemistry to detect levels of pSmad2. Tissue sections were boiled in 10 mm citrate buffer to retrieve endogenous antigens and then exposed to anti-pSmad2 antibody (1:500 dilution, Cell Signaling Technology) overnight. Staining was visualized with 3′,3′-diaminobenzidine as for αvβ6.

Human lung tissue from four fibrotic and four non-fibrotic donors was subjected to immunohistochemistry to detect levels of either αvβ6 (see above for αvβ6) or Elk1. For Elk1, tissue was dewaxed and rehydrated and then boiled in 10 mm citrate buffer. Following blocking in 5% donkey serum, the sections were incubated overnight with either Elk1 antibody (1.1 μg/ml, Abcam clone E277). Staining was visualized using 3′,3′-diaminobenzidine. All staining was visualized using a Nikon 90i light microscope and NIS Elements image acquisition software.

##### Quantification of pSmad2-positive Nuclei

Quantification was performed using a Nikon Eclipse 90i microscope and Nikon NIS Elements software. Five random fields of view at ×20 magnification were observed per tissue section. All brown nuclei present in the field of view were counted.

##### Masson Trichrome Histological Staining

5-μm-thick histological sections of lung tissue were dewaxed and rehydrated using xylene and decreasing concentrations of ethanol. The tissues were incubated overnight at room temperature in Bouin solution. The lung tissue was then subjected to consecutive histological staining in Weigert solution (5 min), Biebrich scarlet acid fuchsin (5 min), phosphomolybolic acid/phototungstic acid (7 min), analine blue (5 min), and 1% acetic acid (1 min). The sections were then dehydrated and imaged using a Nikon Eclipse 90i microscope.

##### Isolation of mRNA from Lung Tissue

Human and murine lung tissue was ground to powder in liquid nitrogen using a pestle and mortar. Frozen lung tissue was then added to TRIzol reagent (1 ml TRIzol/0.5 g of tissue) and incubated on ice for 5 min prior to the addition of chloroform (200 μl/ml TRIzol). The samples were centrifuged at 11,000 × *g* for 20 min to allow phase separation, and then the aqueous layer was transferred to a new tube. RNA was precipitated by addition of 500 μl of isopropyl alcohol and 1 μl of glycogen and centrifuged. The RNA was washed in 75% ethanol prior to being resuspended in nuclease-free water.

##### Hydroxyproline Assay

Hydroxyproline levels in murine lung tissue were measured as described previously (25). Briefly, lung tissue was hydrolyzed in 6 N HCl at 100 °C overnight, and the hydrosylate was resuspended in water. 1.4% chloramine T in 10% isopropanol and 0.5 m sodium acetate were added for 20 min, followed by Erlich solution for 15 min at 65 °C. Absorbance was measured at 550 nm.

##### Human Tissue and Ethical Approval

Lung tissue from pulmonary fibrosis patients (PF) was obtained either post-mortem or from lung transplant patients following informed written consent and ethical review (ethical approval numbers: Nottingham Respiratory Research Unit, 08/H0407/1; Papworth Hospital Research Tissue Bank (REC), 08/H0304/56; University of California San Francisco Institutional Review Board, 10-00198; South East Scotland Scottish Academic Health Sciences Collaboration Bioresource, 06/S1101/41). In all cases, the pathological diagnosis was usual interstitial pneumonia, and the clinical diagnosis of IPF was made on the basis of American Thoracic Society/ European Respiratory Society consensus criteria (26). Non-fibrotic human lung tissue was obtained from non-cancerous tissue removed during surgery or from donor lungs unsuitable for transplant. All experiments were performed in accordance with the World Medical Association Declaration of Helsinki.

Animal studies using bleomycin were approved by the University of Nottingham Ethical Review Committee and performed under Home Office Project and Personal License authority within the Animals (Scientific) Procedures Act of 1986. Animals received free access to food and water at all times.

##### Statistics

All cell-based experiments were repeated three times and expressed as mean data from the three independent experiments. Statistical significance was determined by either *t* test when comparing two data sets or analysis of variance for comparing multiple datasets. Data from experiments comparing PF and control non-fibrotic (NF) human tissues were not normally distributed. Therefore, a non-parametric Mann-Whitney *U* test was used to determine significance. *p* < 0.05 was accepted as significant in all cases.

## Results

### 

#### 

##### The ITGB6 Gene Promoter Contains a Putative Repressor Region

To investigate the presence of transcriptional regulatory regions within the *ITGB6* gene promoter, we created a series of truncated *ITGB6* promoter luciferase reporter constructs from the 1.1-kb *ITGB6* promoter reporter used by others (18) (Fig. 1*A*). With progressively decreasing size, transcription factor binding sites are lost from the reporter constructs. This series of reporter constructs was transfected into iHBECs, and luciferase activity was measured after 4 h. Loss of the most distal regions of the *ITGB6* promoter led to dramatic increases in basal promoter activity (Fig. 1*B*). The increase in basal promoter activity was greatest when the −818 to −731 region of the promoter was absent, suggesting that this region contains important negative regulatory elements. *In silico* analysis using Transcription Element Search Software (University of Pennsylvania) indicated binding sites for several transcription factors within the region −818 to −731. A schematic showing this putative repressor region is shown in Fig. 1*C*.

##### Elk1 Represses ITGB6 and αvβ6 Expression in Vitro

**FIGURE 2.**
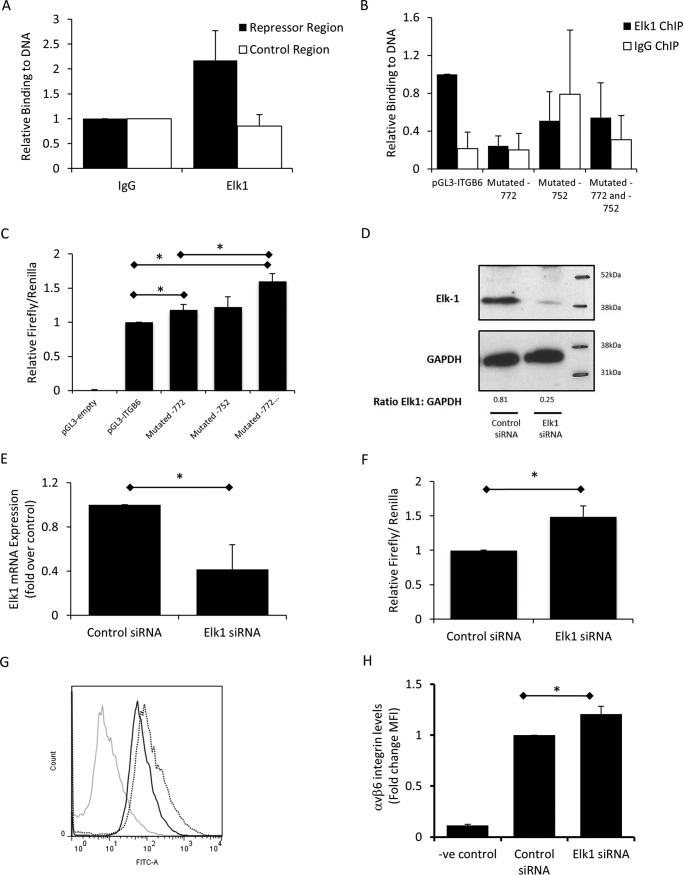
**αvβ6 is repressed by Elk1 binding to the *ITGB6* promoter at −772 and −752.** *A*, basal binding of Elk1 to the repressor region of the endogenous *ITGB6* promoter (−934 to −753) and a control region (−1604 to −1497) was assessed by ChIP in iHBECs. Binding of Elk1 above IgG binding levels was detected at the repressor region but not at the control region. Data are expressed as mean ± S.E. relative binding (relative to IgG) from three independent experiments. *B*, SDM of Elk1 sites located at either −772, −752, or both was performed. H647 cells were transfected with either non-mutated pGL3-*ITGB6* reporter or either of the mutated constructs for 24 h. ChIP was performed for endogenous Elk1. Relative binding above IgG was calculated relative to the full-length, non-mutated promoter construct. Shown is mean relative binding ± S.E. from three independent experiments. *C*, SDM of Elk1 sites located at either −772, −752, or both was performed. iHBECs were transfected with these constructs, and luciferase activity was measured. Data are expressed as mean relative firefly/*Renilla* ratio ± S.E. from three independent experiments. *, *p* < 0.05. *D*, iHBECs were transfected with Elk1 or control siRNA, and Elk1 expression was measured by Western blotting. The figure is representative of three independent experiments. The ratio of Elk1:GAPDH as calculated by densitometry is shown beneath the bands. *E*, iHBECs were transfected with Elk1 or control siRNA, and *ELK1* mRNA was assessed. Data are expressed as mean *ELK1* expression ± S.E. from two independent experiments. *, *p* < 0.05. *F*, iHBECs were cotransfected with Elk1 or control siRNA with the full-length *ITGB6* promoter reporter. Data are expressed as mean relative firefly/*Renilla* ± S.E. from three independent experiments. *, *p* < 0.05. *G*, iHBECs were transfected with either control siRNA or Elk1 siRNA, and αvβ6 expression was assessed after 2 days. *Gray line*, negative control (*-ve*); *black solid line*, control siRNA; *dotted line*, Elk1 siRNA. The histogram is representative of three independent experiments. *H*, amalgamated data from the experiments shown in *G* expressed as -fold change MFI. *, *p* < 0.05.

Previous studies have suggested a role of ets family transcription factors in regulating the *ITGB6* promoter (18). Therefore, we initially investigated the role of Elk1 in *ITGB6* regulation. Using ChIP, we demonstrated that Elk1 bound to the putative repressor region of the *ITGB6* promoter in lung epithelial cells under basal conditions, but binding was not detected at a control region of the promoter upstream of the repressor region (Fig. 2*A*). SDM of Elk1 binding motifs was utilized to investigate the effect of interrupting binding of Elk1 to the *ITGB6* promoter, which contains two Elk1 binding sites within the putative repressor region (at −772 and −752). ChIP was used to assess binding of endogenous Elk1 protein to the exogenous mutated and non-mutated promoter constructs. Binding of endogenous Elk1 to the exogenous, unmutated pGL3-*ITGB6* construct was detected; however, binding above an IgG control was not detected when either Elk1 site was mutated individually or when both sites were mutated simultaneously (Fig. 2*B*).

Following confirmation that Elk1 was unable to bind to the *ITGB6* promoter when the binding sites were mutated, the effect of mutating the Elk1 binding sites on promoter activity was assessed. Mutation of the Elk1 binding site at −772 resulted in a small but significant rise in *ITGB6* promoter activity, whereas mutation of the binding site at −752 had no significant effect (Fig. 2*C*). However, mutation of both binding sites together resulted in a larger increase in *ITGB6* promoter activity, which was greater than the increase observed when the −772 site was mutated alone (Fig. 2*C*).

To determine whether Elk1 repressed *ITGB6* mRNA expression, we utilized an Elk1 siRNA that reduced Elk1 protein and mRNA expression compared with control siRNA (Fig. 2, *D* and *E*). Transfection of Elk1 siRNA increased basal *ITGB6* promoter activity (Fig. 2*F*), suggesting a loss of basal transcriptional repression. Supporting these molecular studies, we demonstrated that Elk1 siRNA also led to a significant increase in αvβ6 cell surface protein levels 2 days following transfection (Fig. 2, *G* and *H*). These data demonstrate that Elk1 regulates *in vitro* lung epithelial cell αvβ6 integrin expression.

##### Glucocorticoid Receptor, but Not Progesterone Receptor, Represses αvβ6 Expression

**FIGURE 3.**
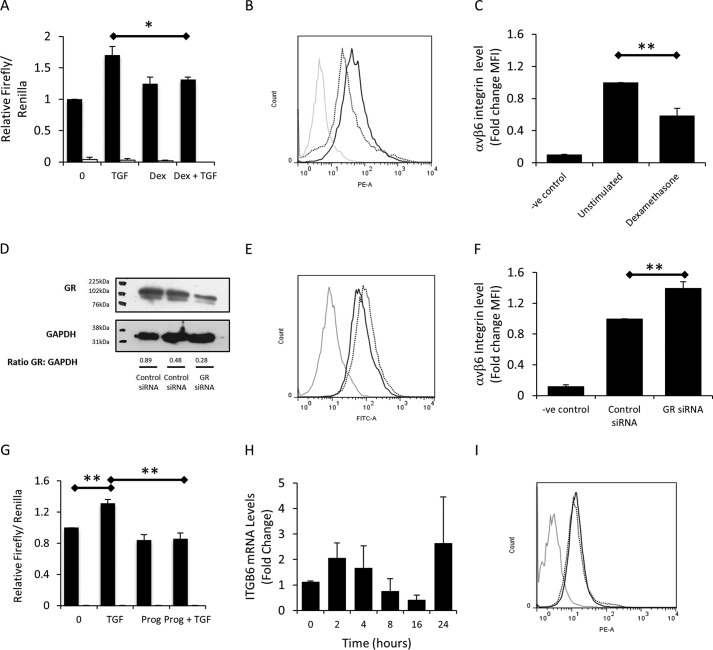
**The glucocorticoid receptor, but not the progesterone receptor, represses αvβ6.** *A*, iHBECs transfected with the *ITGB6* promoter reporter (*black columns*) or an empty vector control (*white columns*) were stimulated with 2 ng/ml TGFβ1, 10 μm dexamethasone (*Dex*), or TGFβ1 and dexamethasone together. Data are expressed as mean relative firefly/*Renilla* ratio ± S.E. from three independent experiments. *, *p* < 0.05. *B*, SAECs were stimulated with dexamethasone for 3 days, and αvβ6 was assessed. *Gray line*, negative control; *solid line*, 0 μm; *dotted line*, 10 μm. The histogram is representative of three independent experiments. *C*, the data shown in *B* expressed as a bar chart. Shown are amalgamated data from three independent experiments expressed as -fold change MFI. **, *p* < 0.01. *D*, iHBECs were transfected with control or GR siRNA, and GR protein expression was determined by Western blotting to confirm GR knockdown. The figure is representative of three independent experiments. The ratio of GR:GAPDH as calculated by densitometry is shown beneath the bands. *E*, iHBECs were transfected with control or GR siRNA, and αvβ6 was assessed after 2 days. *Gray line*, negative control (*-ve*); *black solid line*, control siRNA; *black dotted line*, GR siRNA. The histogram is representative of three independent experiments. *F*, the data shown in *E* expressed as a bar chart. Shown are amalgamated data from three independent experiments expressed as -fold change MFI. **, *p* < 0.01. *G*, iHBECs were transfected with either an *ITGB6* promoter reporter (*black columns*) or an empty vector control (*white columns*) and stimulated with TGFβ1 (2 ng/ml), progesterone (*Prog*, 10 μm), or TGFβ1 and progesterone in combination. Data are expressed as mean relative firefly/*Renilla* ratio ± S.E. from three independent experiments. **, *p* < 0.01. *H*, iHBECs were stimulated with 10 μm progesterone, and *ITGB6* mRNA was measured by QPCR over 24 h. Data are expressed as mean -fold change relative to 0 h ± S.E. from three independent experiments. *I*, SAECs were stimulated with 10 μm progesterone, and αvβ6 was assessed. *Gray line*, negative control; *solid line*, 0 μm; *dotted line*, 10 μm progesterone. The histogram is representative of three independent experiments.

The repressor region identified contains a number of putative transcription factor binding sites (Fig. 1*C*), and the degree of repression mediated by Elk1 was considerably lower than what was observed by deletion of the complete repressor region. We therefore hypothesized that other transcription factors may be contributing to *ITGB6* repression. The receptors for glucocorticoids and progesterone had been identified in the repressor region; therefore, we investigated the effect of these hormones on *ITGB6* expression. Dexamethasone had no effect on the basal activity of the full-length *ITGB6* promoter after 4 h but inhibited TGFβ1-induced increases in *ITGB6* promoter activity in iHBECs (Fig. 3*A*). However, treatment of SAECs with dexamethasone for 3 days resulted in a reduction in cell surface expression of αvβ6 integrins (Fig. 3, *B* and *C*). Additionally, a siRNA targeting GR, which reduced GR protein expression (Fig. 3*D*), caused a small but consistent increase in αvβ6 expression over 2 days (Fig. 3, *E* and *F*). Similar to dexamethasone, progesterone had no significant effect on basal *ITGB6* promoter activity but abrogated TGFβ1-induced increases in promoter activity (Fig. 3*G*). However, progesterone had no significant effect on *ITGB6* mRNA expression over 24 h (Fig. 3*H*) or any effect on αvβ6 cell surface expression in SAECs over 3 days (Fig. 3*I*). These data suggested that, although glucocorticoids may affect *ITGB*6 expression, progesterone was unlikely to be involved in αvβ6 regulation.

**FIGURE 4.**
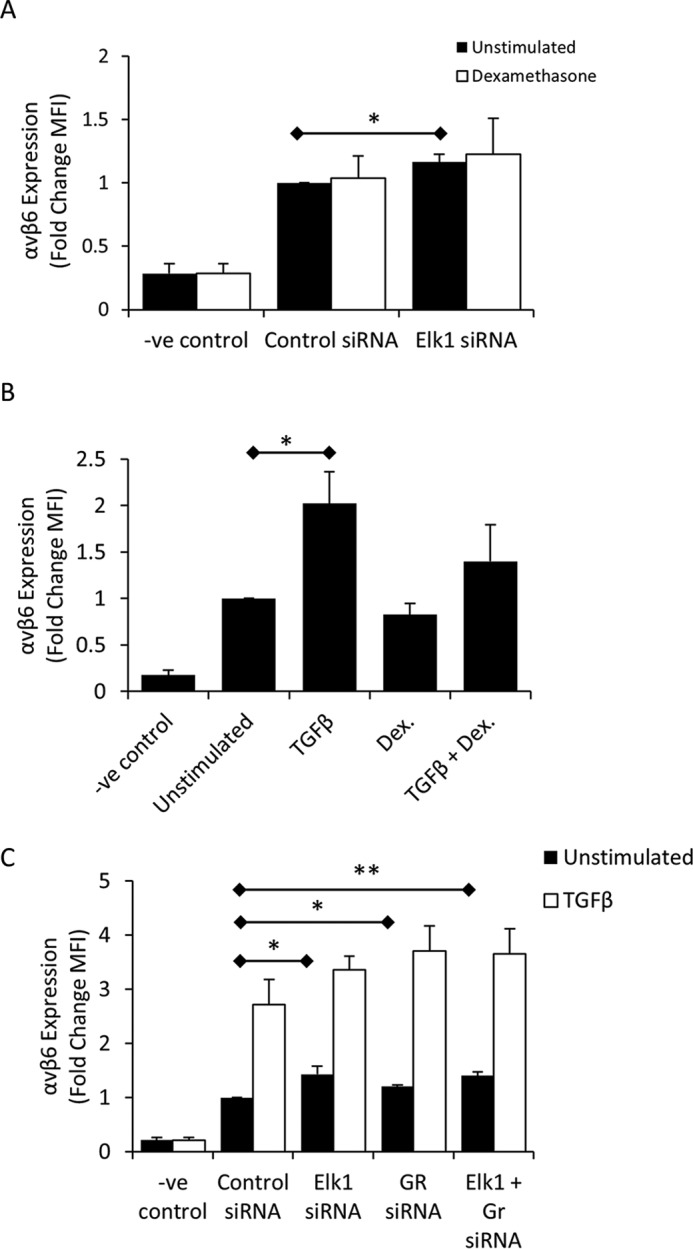
**Inhibiting the actions of Elk1 and GR simultaneously does not have an additive effect on αvβ6 repression.** *A*, iHBECs were transfected with Elk1 siRNA for 24 h and then stimulated with either 0 or 10 μm dexamethasone. αvβ6 expression was determined after 2 days. Shown are amalgamated data from three independent experiments. Data are expressed as -fold change in MFI relative to negative control (*-ve*). *, *p* < 0.05. *B*, iHBECs were left unstimulated or stimulated with 2 ng/ml TGFβ, 10 μm dexamethasone (*Dex*), or both agonists in combination for 3 days. αvβ6 expression was determined by flow cytometry. Shown are amalgamated data from three independent experiments. Data are expressed as -fold change in MFI. *, *p* < 0.05. *C*, iHBECs were transfected with either control siRNA, Elk1 siRNA, GR siRNA, or both siRNAs in combination for 24 h. The cells were then treated with 0 or 2 ng/ml TGFβ for 2 days. αvβ6 expression was measured by flow cytometry. Shown are amalgamated data from three independent experiments. Data are expressed as -fold change in MFI. *, *p* < 0.05; **, *p* < 0.01.

Having identified two transcription factors (Elk1 and GR) capable of binding the putative repressor region and repressing *ITGB6* and αvβ6, we sought to determine whether they co-regulated αvβ6 expression. First, we utilized a combination of dexamethasone treatment to activate GR and Elk1 siRNA and assessed the effect on αvβ6 expression. Confirming the data shown in Fig. 2*G*, transfection of iHBECs with Elk1 siRNA caused a modest but significant increase in αvβ6 expression (Fig. 4*A*). However, activation of the GR with dexamethasone for 2 days did not affect αvβ6 levels in the presence of either control or Elk1 siRNA (Fig. 4*A*). We next investigated the effect of dexamethasone treatment on TGFβ-induced increases in αvβ6 expression. Despite showing a trend toward reducing TGFβ-induced αvβ6 expression, dexamethasone did not have a statistically significant effect on TGFβ-induced increases in αvβ6 expression in iHBECs after 2 days (Fig. 4*B*). Finally, iHBECs were transfected with Elk1 or GR siRNAs or both siRNAs in combination, and αvβ6 expression was measured in unstimulated and TGFβ-stimulated cells. Both Elk1 and GR siRNA increased basal αvβ6 expression, as shown previously, although transfection of both siRNAs did not act synergistically to further enhance αvβ6 cell surface levels (Fig. 4*C*). Transfection with Elk1 and GR siRNA had a similar effect on TGFβ-stimulated cells, although the increase was not statistically significant compared with TGFβ alone (Fig. 4*C*).

##### Loss of Elk1 in Vivo Results in an Exaggerated Response to Bleomycin-induced Lung Fibrosis

**FIGURE 5.**
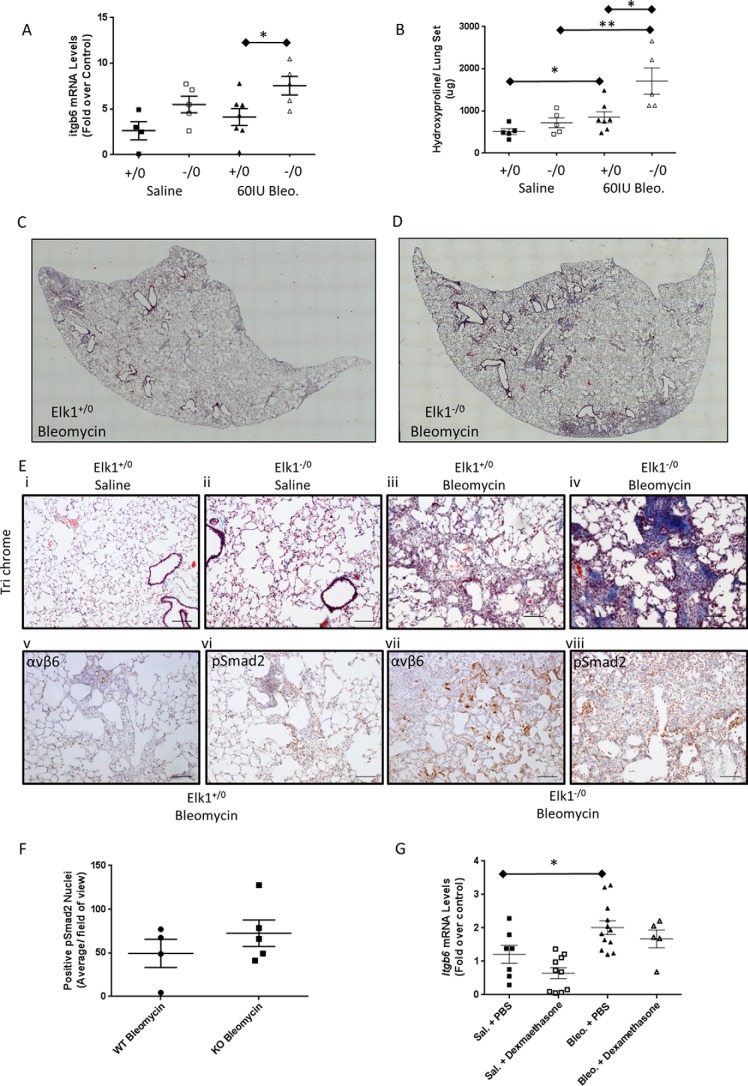
**Loss of *Elk1 in vivo* results in an exaggerated response to bleomycin-induced lung fibrosis.** *A*, *Itgb6* mRNA levels were measured in lung homogenates by QPCR and were significantly elevated in bleomycin (*Bleo*)-instilled *Elk1*^−/^*^0^* mice (*n* = 5 mice) compared with bleomycin-treated *Elk1*^+/^*^0^* control mice (*n* = 7 mice). *, *p* < 0.05. *B*, total lung collagen, assessed by hydroxyproline levels, was significantly elevated in bleomycin-instilled *Elk1*^−/^*^0^* mice (*n* = 5 mice) compared with *Elk1*^+/^*^0^* controls (*n* = 7 mice). *, *p* < 0.05; **, *p* < 0.01. *C*, lung collagen was stained using Masson trichrome. Nikon NIS Elements was utilized to image an entire lung lobe from an *Elk*^+/^*^0^* animal to demonstrate the deposition of collagen across the lobe. Shown is a representative single lung lobe from an *Elk*^+/^*^0^* animal treated with bleomycin (*n* = 4 mice). *D*, lung collagen was stained using Masson trichrome. Nikon NIS Elements was utilized to image an entire lung lobe from an *Elk1*^−/^*^0^* animal to demonstrate the deposition of collagen across the lobe. Shown is a representative single lung lobe from an *Elk1*^−/^*^0^* animal treated with bleomycin (*n* = 4 mice). *E*, histological sections of lung from *Elk1*^−/^*^0^* and *Elk1*^+/^*^0^* animals following a bleomycin model of lung fibrosis were stained with Masson trichrome and for αvβ6 and pSmad2 by immunohistochemistry. All images were acquired using Nikon NIS Elements and are representative of a minimum of three (maximum of five) animals. *Scale bar* = 100 μm. *i*, saline-treated *Elk1*^+/^*^0^* animal stained with Masson trichrome. *ii*, saline-treated *Elk1*^−/^*^0^* animal stained with Masson trichrome. *iii*, bleomycin-treated *Elk1*^+/^*^0^* animal stained with Masson trichrome. *iv*, bleomycin-treated *Elk1*^−/^*^0^* animal stained with Masson trichrome. *v*, bleomycin-treated *Elk1*^+/^*^0^* animal immunostained for αvβ6. *vi*, bleomycin-treated *Elk1*^+/^*^0^* animal immunostained for pSmad2. *vii*, bleomycin-treated *Elk1*^−/^*^0^* animal immunostained for αvβ6. *viii*, bleomycin-treated *Elk1*^−/^*^0^* animal immunostained for pSmad2. *F*, histological sections of lung from *Elk1*^−/^*^0^* and *Elk1*^+/^*^0^* animals following a bleomycin model of lung fibrosis were stained immunohistochemically for pSmad2 (see *E*, *vi* and *viii*). pSmad2-positive nuclei were quantified. Data are expressed as mean brown nuclei per field of view in bleomycin-treated Elk1^+/^*^0^* (*n* = 4) and *Elk1*^−/^*^0^* (*n* = 5) animals. *G*, animals were instilled with either bleomycin or saline (*Sal*) and treated with either PBS or dexamethasone daily. *Itgb6* mRNA levels were measured in lung homogenates by QPCR after 14 days and were significantly elevated in bleomycin-instilled, PBS-treated mice (*n* = 12 mice) compared with saline-instilled, PBS-treated mice (*n* = 7 mice). *, *p* < 0.05.

Although the *in vitro* data suggested that Elk1 was unlikely to mediate complete repression of the *ITGB6* promoter, we hypothesized that even moderate effects on epithelial αvβ6 integrin expression were likely to be important in response to injury. Therefore, to determine whether global loss of Elk1 was sufficient to increase *Itgb6* expression *in vivo* and exacerbate fibrotic responses, we employed the bleomycin model of lung fibrosis. *Elk1*^−/^*^0^* male mice and wild-type littermate controls (*Elk1*^+/^*^0^*) were instilled with saline or 60 IU of bleomycin. Lungs were harvested 28 days following bleomycin instillation, and then lung fibrosis and *Itgb6* mRNA levels were measured. Bleomycin increased the levels of *Itgb6* mRNA in homogenates of Elk1^−/^*^0^* and *Elk1*^+/^*^0^* whole lung tissues. However, there were significantly greater levels of *Itgb6* mRNA in the lungs of *Elk1*^−/^*^0^* animals treated with bleomycin (Fig. 5*A*).

Lung fibrosis was quantified by measuring levels of hydroxyproline in lung tissue and by assessing histology using Masson trichrome stain. Bleomycin treatment increased lung hydroxyproline levels in both *Elk1*^+/^*^0^* and *Elk1*^−/^*^0^* animals. However, lungs from bleomycin-treated *Elk1*^−/^*^0^* mice contained significantly higher levels of hydroxyproline compared with bleomycin-treated *Elk1*^+/^*^0^* animals (Fig. 5*B*). To confirm that deposition of collagen in the lungs in response to bleomycin was greater in *Elk1*^−/^*^0^* animals compared with control animals, histological sections of lung tissue were stained with Masson trichrome. Low-power, stitched images of entire lung lobes demonstrate the patchy nature of lung fibrosis following bleomycin-induced lung injury but show that the areas of fibrosis were considerably larger in the *Elk1*^−/^*^0^* animals compared with control animals (Fig. 5, *C* and *D*), and this was confirmed in high-power images (Fig. 5*E*, *i–iv*). Lung tissue from these animals was also stained by immunohistochemistry for αvβ6 integrins. There was no evidence of αvβ6 protein expression in lungs of injured mice (data not shown), consistent with previous reports (25). Although expression of αvβ6 integrin was evident in areas of lung damage in both *Elk1*^−/^*^0^* and *Elk1*^+/^*^0^* animals, expression was markedly increased in *Elk1*^−/^*^0^* compared with wild-type control animals (Fig. 5*E*, *v* and *vii*). Furthermore, *Elk1*^−/^*^0^* animals demonstrated a trend toward increased pSmad2 staining compared with *Elk1*^+/^*^0^* animals (Fig. 5, *E*, *vi* and *viii*, and *F*), consistent with reduced Elk1 expression, leading to enhanced expression of TGFβ-activating integrins. Taken together, these data support the hypothesis that Elk1 is capable of suppressing *Itgb6* mRNA and αvβ6 expression *in vivo* and ultimately limit the fibrotic response to injury *in vivo*.

Having identified that GR could also repress *ITGB6 in vitro*, we evaluated its effect *in vivo*. Animals were treated with bleomycin to induce lung injury and *Itgb6* expression or with saline and received daily intraperitoneal injections of either dexamethasone or a PBS control. *Itgb6* mRNA levels were determined in the lungs after 14 days. There was a clear trend toward reduced *Itgb6* expression in both the saline and bleomycin groups treated with daily dexamethasone compared with the groups treated with daily PBS (Fig. 5*G*), but this did not reach statistical significance. However, when the data were analyzed to determine any effect of dexamethasone irrespective of the presence of lung injury, there was a significant reduction in *Itgb6* mRNA following dexamethasone treatment compared with PBS treatment (*p* = 0.01). This effect was also observed after 21 days of treatment (data not shown). These data confirm the *in vitro* observations and show that Elk1 and GR can regulate expression of *Itgb6* in murine lung.

##### The Repressor of ITGB6 Transcription, Elk1, Is Reduced in Pulmonary Fibrosis

**FIGURE 6.**
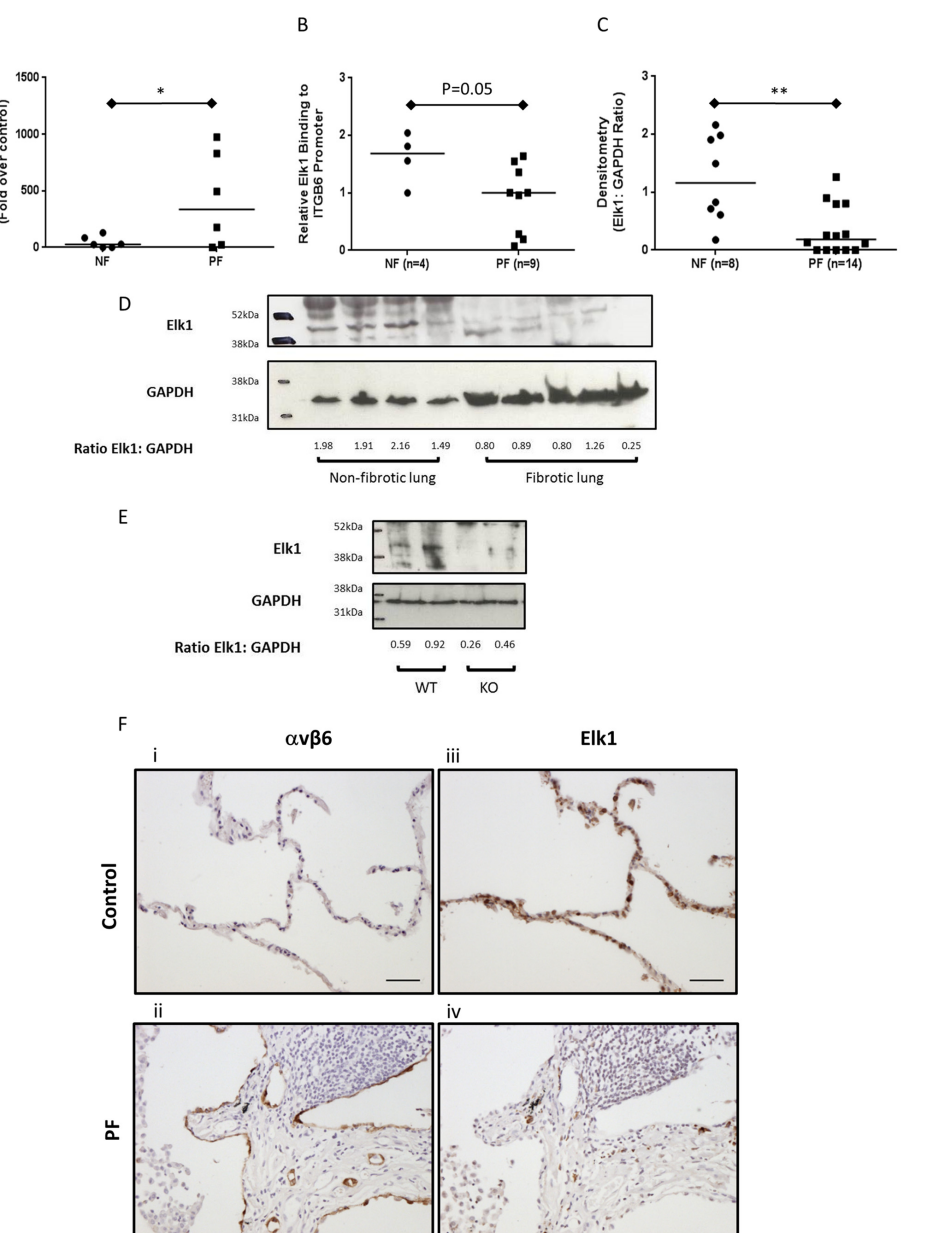
**Expression of Elk1 is reduced in pulmonary fibrosis.** *A*, relative expression of *ITGB6* mRNA in NF (*n* = 6 donors) and PF (*n* = 6 donors) human lung tissue was assessed by QPCR. Data are expressed as relative mRNA expression compared with NF samples. The *line* demonstrates the median. *, *p* < 0.05. *B*, binding of Elk1 to the *ITGB6* promoter in NF and PF tissue as measured by ChIP. Data are expressed as relative binding to the *ITGB6* promoter compared with the IgG control, and the median is expressed. Relative binding of 1 or below demonstrates no binding to the promoter above IgG. *C*, densitometry analysis of Elk1 expression in whole-lung extracts from NF (*n* = 8 donors) and PF (*n* = 14 donors) donors. Data are expressed as a ratio of Elk1 band intensity:GAPDH band intensity. **, *p* < 0.01. *D*, representative immunoblot showing Elk1 and GAPDH expression in NF (*n* = 4 donors) and PF (*n* = 5 donors) lung tissue. The ratio of Elk1:GAPDH as calculated by densitometry is shown beneath the bands. *E*, immunoblot showing Elk1 and GAPDH protein expression in Elk1^+/^*^0^* (*n* = 2 mice) and *Elk1*^−/^*^0^* (*n* = 2 mice) animals. The ratio of Elk1:GAPDH as calculated by densitometry is shown beneath the bands. *F*, histological sections of lung from control (*n* = 4 donors) and PF (*n* = 4 donors) donors were immunostained for αvβ6 and Elk1. All images were acquired using Nikon NIS Elements and are representative of the donor samples used. *Scale bar* = 100 μm. *i*, lung tissue from a control donor was stained for αvβ6. *ii*, lung tissue from a PF donor was stained for αvβ6. *iii*, lung tissue from a control donor was stained for Elk1. *iv*, lung tissue from a PF donor was stained for Elk1.

Having identified that Elk1 is important for repression of *ITGB6* expression in the lung, we hypothesized that this pathway would be dysregulated in human disease. We have previously demonstrated that *ITGB6* mRNA levels are increased in lung tissue homogenates from lung fibrosis patients (25), which we again found in this study (Fig. 6*A*). ChIP analysis demonstrated that there was binding of Elk1 to the *ITGB6* promoter in non-fibrotic human lung tissue and that there was reduced binding in samples from patients with PF (*p* = 0.05, Fig. 6*B*). It was clear that, in some patients with PF, there was no detectable binding of Elk1 to the *ITGB6* promoter above IgG controls (Fig. 6*B*). Therefore, levels of Elk1 protein were assessed by Western blotting in PF and NF lung tissue samples. Elk1 was expressed in all control samples tested (Fig. 6, *C* and *D*), but the levels were lower, and in some cases undetectable, in the PF tissue tested. Quantification of the Western blotting signals by densitometry demonstrated that PF patients had significantly less Elk1 protein than controls (Fig. 6*C*). The specificity of the antibody used was confirmed by probing protein samples from both wild-type and Elk1^−/^*^0^* animals. Elk1 protein was detected in wild-type animals but not Elk^−/^*^0^* animals (Fig. 6*E*).

To assess expression of Elk1 protein macroscopically in human lung tissue, serial sections of paraffin wax-embedded lung tissue from NF (*n* = 4) and PF (*n* = 4) donors were immunostained for both αvβ6 and Elk1. Confirming previous reports (13), NF donors had low levels of αvβ6 expression within the alveolar epithelium (Fig. 6*F*, *i*). In contrast, lung tissue from PF donors demonstrated marked expression of αvβ6 integrins within the alveolar epithelium that was particularly evident around regions of fibrosis (Fig. 6*F*, *ii*). Supporting a role for Elk1 being responsible for negative regulation of αvβ6 integrins and confirming that Elk1 protein expression is aberrant in fibrosis, we found that NF donors had high levels of epithelial Elk1 protein expression, whereas PF donors had minimal expression of Elk1 within the lung epithelium (Fig. 6*F*, *iii* and *iv*).

## Discussion

IPF is a progressive lung disease with extremely poor survival and no effective treatments. Activation of TGFβ via αvβ6 integrins, the expression of which is limited to epithelial cells, is a fundamental process in the pathogenesis of pulmonary fibrosis (7, 9, 11). Interactions between lung epithelial cells and fibroblasts, partly through epithelial cell αvβ6-mediated TGFβ activation, are now thought to be critical to the development and progression of lung fibrosis (4). A common observation in IPF patients and in other fibrotic disorders is the up-regulation of αvβ6 integrins within the epithelium (9, 12, 14, 15). The molecular mechanisms driving enhanced αvβ6 expression, however, have not been fully investigated. In this study, we highlighted a region of the *ITGB6* gene promoter responsible for repressing basal gene transcription and, fundamentally, identified a novel and important function for the transcription factor Elk1 in fibrosis as a negative regulator of *ITGB6* and αvβ6 integrins in epithelial cells.

Previous studies have suggested that recombinant Elk1 reduces activity of an exogenous *ITGB6* promoter in the HEK293 cell line (18). Our data are consistent with these findings and demonstrate that loss of Elk1 or interruption of the ability of Elk1 to bind to the *ITGB6* promoter increases *ITGB6* promoter activity in lung epithelial cells, which can, in turn, lead to increased expression of αvβ6 integrins *in vitro*. Furthermore, loss of Elk1 *in vivo* increased levels of *Itgb6* mRNA and collagen deposition in a bleomycin model of lung fibrosis. Elk1 is a ternary complex factor that is commonly thought of as an activator of transcription when phosphorylated. However, it also contains an R domain that can potently repress transcription (27). The role of Elk1 phosphorylation has not yet been determined on *ITGB6* expression and may prove to be an important regulatory pathway. It is possible that dynamic regulation between unphosphorylated Elk, which functions as a transcriptional repressor, and phosphorylated Elk1, which can act as an transcriptional activator, may be important in repair processes and is the focus of current studies.

Other putative repressors of the *ITGB6* promoter highlighted by *in silico* analysis included the progesterone receptor and GR. Both the progesterone receptor and GR are expressed in the normal lung (28, 29), and, although the effects of progesterone on *ITGB6* expression have not been investigated previously either in the lung or in other tissues, it has been shown to modulate the expression of several other integrin subunits, including the α2 and α11 subunits, as well as α1β1 and αvβ3 integrins in the placenta and endometrium (30, 31). However, we found no convincing effect of progesterone on basal *ITGB6* promoter activity, *ITGB6* mRNA levels, or cell surface αvβ6 integrin levels, suggesting that the progesterone receptor is not a major contributor to repression of *ITGB6* in lung epithelial cells.

In contrast with progesterone, dexamethasone was able to inhibit TGFβ1-induced *ITGB6* promoter activity and basal αvβ6 integrin cell surface expression and showed a trend toward reducing TGFβ-induced αvβ6 expression *in vitro*. Furthermore, confirming our *in vitro* data, activation of GR *in vivo* using daily treatment with dexamethasone led to reduced expression of *Itgb6* mRNA in the lung. Although it is possible that the inhibition of αvβ6 integrin expression is a direct result of GR activation with consequent binding to, and repression of, the *ITGB6* promoter, we do not favor this hypothesis. Our data suggest that dexamethasone does not affect basal promoter activity but, rather, inhibits TGF-β1-induced *ITGB6* expression. Furthermore, it has previously been shown that GR can interrupt TGFβ/Smad3-mediated plasminogen activator inhibitor 1 (PAI1) expression via a physical interaction with the transcriptional activation functions of Smad3 (32). Because TGF-β has been postulated as a positive regulator of αvβ6 integrins via an autocrine loop (17), we hypothesize that GR reduces αvβ6 protein expression via interrupting this TGF-β-mediated autocrine loop.

The effect of siRNA-induced knockdown of Elk1 on both *ITGB6* promoter activity and αvβ6 integrin expression was relatively modest, and the effect of Elk1 siRNA does not completely recapitulate the effect of loss of the entire repressor region from the *ITGB6* promoter. Efficiency of the siRNA transfection is unlikely to have reached 100%, which is supported by the observation that transfection with Elk1 siRNA did not result in 100% knockdown of Elk1 protein or mRNA. Furthermore, the process of transfecting cells *in vitro* can often result in cell injury (33, 34), and it is well documented that epithelial cell injury results in up-regulation of αvβ6 (8, 12), which raises the possibility that transfection of the control siRNA might have increased αvβ6 expression, therefore reducing the effect “window” observed when comparing Elk1 siRNA with control siRNA. Unfortunately, the nuclear location of Elk1 made alternative methods of inhibiting Elk1 difficult.

Importantly, the repressor region of the *ITGB6* promoter contains multiple transcription factor binding sites, many of which may also exhibit repressor functions on the *ITGB6* gene. In addition to Elk1, our data highlight a potential role for GR in suppressing αvβ6 expression *in vitro* and *Itgb6 in vivo*. However, inhibiting the actions of Elk1 and GR simultaneously did not have co-regulatory effects on αvβ6 expression over and above inhibiting either transcription factor singularly. It is, therefore, very likely that other factors that may bind the repressor region are involved in the basal repression of the *ITGB6* gene, some of which may have combined additive effects on αvβ6 repression. However, it is beyond the scope of this manuscript to investigate them all. Fundamentally, a consistent effect of manipulating Elk1 on *ITGB6* promoter, gene, and protein expression was observed both *in vitro* and *in vivo*, highlighting a novel and important function for Elk1 in *ITGB6* regulation.

These data are the first description of aberrant Elk1 expression in any fibrotic condition and could help explain the mechanisms driving enhanced αvβ6 expression in IPF. Elk1 is an X-linked gene. Thus, defects in a single allele will have greater effects in males, which may potentially explain the gender differences associated with IPF (35). Levels of Elk1 protein were reduced in lung tissue homogenates and histological sections from PF patients, and, furthermore, tissue ChIP analysis revealed that Elk1 binding to the *ITGB6* promoter was reduced in PF tissue. Expression of *ITGB6* is restricted to the epithelium; therefore, these data suggest that a defect in total lung Elk1 is likely to play a role in the derepression of epithelial αvβ6 integrin expression observed in fibrotic lung tissue. Although the mechanisms regulating decreased Elk1 expression in pulmonary fibrosis are unclear, Elk1 is a target of mir-185, which is increased in lung tissue from patients suffering from rapidly progressive IPF (36).

Although the focus of this manuscript is on the effect of Elk1 on expression of epithelial restricted αvβ6 integrins, it is apparent that global loss of Elk1 expression in IPF could have implications for multiple pathways in many other cells types involved in the pathogenesis of IPF. Elk1 signaling has been implicated in regulating elastin expression in lung fibroblasts (37), hyaluronan expression in dermal fibroblasts (38), and α-smooth muscle actin in vascular smooth muscle cells (39), all of which could have implications for fibrogenesis. Our own *in silico* analysis has highlighted the presence of Elk1 binding motifs in the promoters of several fibrotic genes, including α-smooth muscle actin, the β8 integrin subunit, and TGFβRII, among others. Loss of Elk1 may also affect the expression of other integrins, such as the αvβ1 integrin, which has recently been implicated in fibrogenesis in multiple organs (40).

We propose a novel function for the transcription factor Elk1 in fibrosis. Following epithelial cell injury, Elk1 serves to limit the repair response through repression of *ITGB6* transcription and, therefore, αvβ6 integrins. Where there is failure of repression through, in part, loss/interruption of Elk1 expression/signaling, fibrogenesis may ensue through enhanced and sustained expression of *ITGB6.* In summary, this study describes a novel function for the transcription factor Elk1 in contributing to repression of αvβ6 integrins in the pulmonary epithelium and show that reduced Elk1-mediated repression is important in driving the increased αvβ6 integrin expression and collagen deposition observed in pulmonary fibrosis.

## Author Contributions

A. L. T. conceived and coordinated the study, performed all experiments except those listed below, and wrote the manuscript. A. H. assisted with conducting the *in vivo* animals studies, and performed the histochemical staining of mouse lung tissue. J. P. performed the immunohistochemical staining of human lung tissue shown in Fig. 5. A. E. J. provided immunohistochemical and microscopy expertise for obtaining the data in Figs. 4 and 5. A. S. assisted with the *in vivo* animal studies. E. H. conducted the SDM to produce ITGB6 promoter constructs with mutated Elk1 binding sites. C. K. L. performed the progesterone promoter studies shown in Fig. 2. S. M. V. and P. H. W. created and provided the αvβ6 blocking antibody and contributed to manuscript preparation. A. J. K., G. L., H. P., and P. J. W. were involved in the experimental design and critique of the manuscript. W. W. contributed to the experimental design and manuscript critique and provided the human lung tissue from IPF patients. S. A. and A. N. were involved in the studies using Elk1-null animals (Fig. 4). G. J. conceived the study and wrote the manuscript. All authors approved the final version of the manuscript.

## Footnotes

2 The abbreviations used are:

IPF: idiopathic pulmonary fibrosis
GR: glucocorticoid receptor
iHBEC: immortalized human bronchial epithelial cell
SAEC: small airway epithelial cell
QPCR: quantitative PCR
SDM: site-directed mutagenesis
PF: pulmonary fibrosis
NF: non-fibrotic
MFI: mean fluorescence intensity.
